# Amyloid PET, FDG-PET or MRI? - the power of different imaging biomarkers to detect progression of early Alzheimer’s disease

**DOI:** 10.1186/s12883-019-1498-9

**Published:** 2019-10-31

**Authors:** Marion Ortner, René Drost, Dennis Heddderich, Oliver Goldhardt, Felix Müller-Sarnowski, Janine Diehl-Schmid, Hans Förstl, Igor Yakushev, Timo Grimmer

**Affiliations:** 1 Department of Psychiatry and Psychotherapy, School of Medicine, Klinikum rechts der Isar, Technical University of Munich, Ismaninger Str. 22, 81675 Munich, Germany; 2 Department of Diagnostic and Interventional Neuroradiology, School of Medicine, Klinikum rechts der Isar, Technical University of Munich, Ismaninger Str. 22, 81675 Munich, Germany; 3 Department of Nuclear Medicine, School of Medicine, Klinikum rechts der Isar, Technical University of Munich, Ismaninger Str. 22, 81675 Munich, Germany

**Keywords:** PiB-PET, FDG-PET, MRI, Cortical thickness, Effect size, Minimal sample size, Serial follow-up

## Abstract

**Background:**

As investigations of disease modifying drugs aim to slow down progression of Alzheimer’ disease (AD) biomarkers to reliably track disease progression gain more importance. This is especially important as clinical symptoms, including psychometric measures, are only modestly associated with the underlying disease pathology, in particular at the pre-dementia stages. The decision which biomarkers to choose in clinical trials is crucial and depends on effect size. However, longitudinal studies of multiple biomarkers in parallel that allow direct comparison on effect size are scarce.

**Methods:**

We calculated effect size and minimal sample size for three common imaging biomarkers of AD, namely amyloid deposition measured with PiB-PET, neuronal dysfunction measured with FDG-PET and cortical thickness measured with MRI in a prospective 24-month follow-up study in a monocentric cohort of early AD.

**Results:**

Post hoc power calculation revealed large effect sizes of Cohen’s d for PiB-PET and cortical thickness and a small effect size for FDG-PET (1.315, 0.914, and 0.341, respectively). Accordingly, sample sizes for PiB-PET and cortical thickness required significantly smaller sample sizes than FDG-PET to reliably detect statistically significant changes after 24 months in early AD (*n* = 7, *n* = 12, and *n* = 70, respectively).

**Conclusion:**

Amyloid imaging with PET and measuring cortical thickness with MRI are suitable biomarkers to detect disease progression in early AD within a small sample.

## Background

While there is still no definite treatment for the cause of Alzheimer’s disease (AD), modifying therapies that aim to slow down disease progression are under investigation. Primary endpoints in these trials are usually measures of cognitive functions such as the Alzheimer’s Disease Assessment Scale - Cognitive Subscale (ADAS-cog) [1] or the Clinical Dementia Rating Scale (CDR) [2–5]. However, the progression of clinical symptoms varies among patients with AD and is not always closely associated with the progression of underlying disease, namely beta-amyloid (Aβ) and tau deposition in conjugation with synaptic and neuronal loss [6]. The presentation of clinical symptoms could be modified by brain cognitive reserve capacity [7] and other factors, for example ceiling and floor effects of psychometric tests such as the ADAS-Cog [8]. Thus, to assess the efficacy of disease modifying therapies, biomarkers reflecting the characteristic histopathological features of AD might be more suitable than psychometric tests [9]. Biomarkers for Aβ accumulation are positive uptake of an amyloid tracer, such as ^11^C-Pittsburgh compound B (PiB), on positron emission tomography (PET) and decreased Aβ1–42 in cerebrospinal fluid (CSF) [6]. Biomarkers for tau pathology include tau PET and CSF p-tau. Biomarkers for neuronal dysfunction include specific regions with reduced ^18^F-fluordeoxyglucose (FDG) uptake on PET, and for neuronal loss atrophy on magnetic resonance imaging (MRI) and elevated t-tau protein in CSF [6]. The vast majority of these biomarkers have been studied in terms of specificity, validity, change over time, and correlation with clinical symptoms and other biomarkers [10–18]. However, there are only very few long-term follow-up studies investigating more than two biomarkers at multiple time points within the same patient cohort and not all biomarkers were available for all subjects in these studies [19–21]. To our knowledge, no previous study investigated effect sizes and minimal samples sizes to detect changes of these biomarkers within one monocentric cohort over time.

In this long-term predefined 24-month follow-up study we set out to determine effect sizes and minimal sample sizes for three imaging biomarkers in the same monocentric Alzheimer’s disease cohort: Aβ deposition measured with PiB-PET, neuronal dysfunction measured with FDG-PET, and neuronal loss measured by cortical thickness on MRI.

## Methods

### Ethics statement

The study protocol was approved by the German radiation protection authority and the ethics committee of the School of Medicine of the Technical University of Munich, Munich, Germany (reference number 1285/05). All patients provided written informed consent prior to any study-specific procedures and all clinical investigations were conducted in accordance with the principles of the Declaration of Helsinki.

### Patient recruitment and study design

Patients were recruited from the outpatient clinic of the Centre for Cognitive Disorders at the Department of Psychiatry, Klinikum rechts der Isar, Technical University of Munich, School of Medicine, Munich, Germany. They underwent a standardized diagnostic procedure including a neuropsychological evaluation as described previously [22]. This workup included an interview with the patient and an informant, obtaining demographic data, medical history, and concomitant medication as well as physical, neurological, and psychiatric examinations, a neuropsychological evaluation including the Mini-Mental State Examination (MMSE) [23], the Consortium to Establish a Registry for Alzheimer’s Disease Neuropsychological Assessment Battery (CERAD-NAB) [24], a routine laboratory screening test, and Apolipoprotein E genotyping [25]. The severity of cognitive impairment was rated on the Clinical Dementia Rating scale sum of boxes (CDR-SOB) [26]. It is calculated by adding the ratings of the individual domains of the CDR, resulting in a sum-score between 0 and 18. This allows for a finer gradation and monitoring of symptom progression than using the CDR-global score which gives information about the over-all severity of dementia on a scale between 0 and 3 [27, 28]. Accordingly, while changes in individual categories increase the CDR-SOB score the CDR-global score may remain unchanged. As a consequence, associations between clinical worsening of dementia and changes of individual biomarkers might be missed.

Imaging procedures included cranial MRI to assess structural brain abnormalities and cortical thickness, cerebral FDG-PET to determine neuronal metabolic deficit, and cerebral PiB-PET to assess brain amyloid deposition. All baseline assessments were completed within 1 month for each subject. Follow-up assessments were planned to take place 24 months after the initial evaluation.

In order to participate in this study, subjects needed to have AD typical hypometabolism on FDG-PET [29], as well as positive PiB uptake on visual analysis of the scans. Scans were evaluated by an experienced nuclear medicine specialist. All study participants met the National Institute on Aging and Alzheimer’s Association (NIA-AA) diagnostic criteria for early Alzheimer’s disease [6, 30], and can be placed in the Alzheimer’s continuum (A + T*(N)+: evidence of abnormal biomarkers for Aβ and neuronal injury; a biomarker for tau is not available as indicated by the asterisk) [31]. CDR-sum of boxes (SOB) scores of the subjects ranged between 0.5–9.0.

Exclusion criteria were described previously [22] and included other neurologic or psychiatric disorders, major morphologic or vascular MRI abnormalities, and patients with other possible causes of cognitive impairment such as psychotropic medication or major abnormalities in routine blood testing.

### Brain imaging

Patients underwent cranial **MRI** examinations on a Siemens 1.5 Tesla Magnetom Symphony scanner using a standardized imaging protocol which consisted of a 3D T1 dataset (TR 1520 ms, TE 3.93 ms, 256 × 256 matrix, flip angle 15°, 1 mm slices), axial T2 weighted turbo-spin-echo images (TR 4510 ms, TE 104 ms, 19 slices, voxel dimensions 0.6 mm × 0.5 mm × 6.0 mm), coronal T1 weighted spin echo images (TR 527 ms, TE 17 ms, 19 slices, voxel dimensions 0.9 mm × 0.9 mm × 6.0 mm), T2 weighted gradient echo images (TR 725, TE 29, 19 slices, voxel dimensions 0.7 mm × 0.7 mm × 6.0 mm), and axial FLAIR images (TR 9000 ms, TE 105 ms, TI 2500 ms, 3 mm slices). Datasets were normalized to the MRI MNI-template in SPM8 to collect warping parameters for PET images. **FDG-PET** images were obtained using a Siemens ECAT HR+ PET scanner (CTI, Knoxville, Tenn., USA). Subjects received 370 MBq FDG at rest with eyes closed. Patients were positioned with the head parallel to the canthomeatal line within the gantry. Thirty minutes after injection, PET imaging was performed under standard resting conditions (eyes closed in dimmed ambient light). A sequence of one frame of 10 min and two frames of 5 min was started and later summed into a single frame. Image data were acquired in 3D mode with a total axial field of view of 15.5 cm. A transmission scan was acquired after completion of the emission scan for attenuation correction [32]. **PiB-PET** examinations were performed on the same scanner and followed a standardized protocol [33]. All patients were injected with 370 MBq PiB at rest outside the scanner. 30 min later they were placed in the scanner. At 40 mininutes post-injection, three 10-min frames of data acquisition were started and later summed into a single frame (40–70 min). Acquisition was carried out in 3D mode, and a transmission scan was carried out to allow for later attenuation correction.

The PiB and the FDG images were co-registered and normalized to the MNI space using the warping parameters of the MRI and smoothed using a Gaussian kernel of 10 mm × 10 mm × 10 mm [34]. PiB-PET, FDG-PET, and MRI of the brain were processed according to previously described standardized protocols using statistic parametric mapping software 8 (SPM 8 Wellcome Department of Cognitive Neurology, London, UK) in MATLAB 12 (The MathWorks, Inc., Natick, Massachusetts, USA) and FreeSurfer software Stable release version 5.1.0 [10, 14, 32, 34–37] (http://surfer.nmr.mhg.harvard.ed/; http://www.freesurfer.net/fswiki/VolmeRoiCorticalThickness).

### Statistical analyses

To control for between-subjects differences in PiB uptake, standardized uptake value ratios (SUVRs) were obtained by calculating the cerebral to cerebellar vermis (C/cv) ratio for each patient as demonstrated previously [10, 34]. SUVRs of FDG uptake in AD signature regions [38] were similarly obtained with the exception that the pons was used as reference region (C/pons) [10]. The anatomical ROIs were defined using an established template [37]. For both PET modalities mean standard uptake volume values were calculated for 100% of voxels for each ROI.

Global cortical thickness score per participant was calculated using the averaged cortical thickness values as defined in the Desikan-Killiany-Tourville (DKT) protocol [39] in FreeSurfer software as described previously [36].

Differences between baseline and follow-up examination for all patients, irrespectively of the clinical stage within the AD continuum, were calculated for PiB uptake, FDG uptake, cortical thickness, CDR-SOB and MMSE and adjusted to a 24 months follow-up period.

Based on mean values and standard deviations post-hoc effect sizes (Cohen’s d) and minimal sample sizes (n) to detect a statistically significant change (two-tailed test, α = 5%, β = 20%) of each biomarker were calculated using G*power 3.1 [40, 41].

## Results

### Patients

The early AD patient sample is described in Table 1.

**Table 1:** Patients characteristics

Number of subjects (n)	17
Male / Female	10 (58.8%) / 7 (41.2%)
Early AD (MCI due to AD / mild dementia due to AD)	9 (52.9%) / 8 (47.1%)
	Mean ± SD (Min – Max)
Age at BL [years]	66.76 ± 6.34 (55–77)
Time to follow-up [months]	26.59 ± 2.21 (23–30)
CDR-global (BL)	0.70 ± 0.25 (0.5–1)
ΔCDR-global after 24 months	0.46 ± 0.51 (0.5–2)
CDR-SOB (BL)	4.09 ± 2.12 (0.50–9.00)
ΔCDR-SOB after 24 months	2.29 ± 2.74 (− 2.09–6.92)
MMSE (BL)	23.65 ± 3.39 (16–28)
ΔMMSE within 24 months	−4.64 ± 4.59 (− 13.33–2.88)
ApoE ε4 allele carrier status Homozygous/ heterozygous/ non-carrier	3 / 8 / 6

*MCI* Mild cognitive impairment, *AD* Alzheimer’s disease, *SD* Standard deviation, *min* Minimum, *max* Maximum, *BL* Baseline, *CDR-SOB* Clinical dementia rating scale-sum of boxes, *Δ* Changes between baseline and follow up adjusted to a 24 months follow up period, negative values indicate decrease, positive values indicate increase compared to BL, *MMSE* Mini-mental state examination, *ApoE* Apolipoprotein E

### Effect sizes of imaging biomarkers and minimal sample sizes

**Fig. 1:**
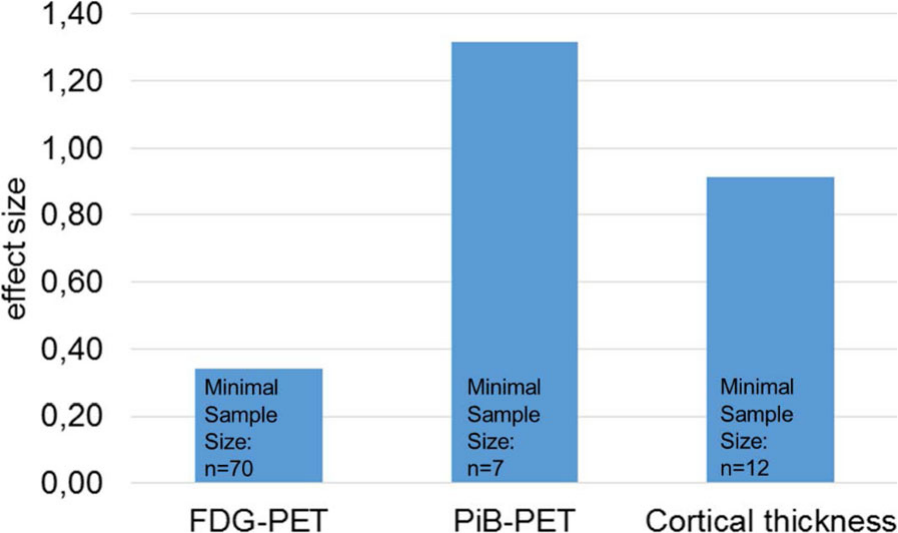
Effect sizes and minimal sample sizes based on post-hoc power calculations for the individual biomarkers to monitor disease progression in early AD in a prospective 24-month follow-up study. n: number; FDG-PET: ^18^F-fluordeoxyglucose positron emission tomography; PiB-PET: ^11^C-Pittsburgh compound B (PiB)

Post hoc power calculation for PiB-PET, FDG-PET, and cortical thickness revealed effect sizes of Cohens’s d = 1.315, 0.341, and 0.914, respectively. Accordingly, sample sizes of at least *n* = 7 for PiB-PET, *n* = 70 for FDG-PET, and *n* = 12 for cortical thickness are required to detect statistically significant changes of AD imaging biomarkers over time with a power of 80% (α = 5%, β = 20%). Results of global imaging data and power analyses for effect sizes and minimum sample sizes are shown in Table 2 and Fig. 1.

**Table 2:** Biomarker characteristics

	Mean ± SD (Min - Max)	Effect size (Cohen’s *d*)	Minimal sample size (*n*)
FDG AD-ROIs/pons (BL)	1,28 ± 0.15 (1.01–1.51)		
Δ (FDG AD-ROIs/pons) within 24 months	−0.05 ± 0.15 (− 0.37–0.32)	0.341	70
PiB global/cerebellar vermis (BL)	1.63 ± 0.22 (1.37–2.16)		
Δ (PiB global/cerebellar vermis) within 24 months	0.12 ± 0.09 (− 0.05–0.25)	1.315	7
Cortical thickness global (BL) [mm]	2.31 ± 0.23 (1.87–2.80)		
Δ Cortical thickness global within 24 months [mm]	−0.14 ± 0.15 (− 0.45–0.11)	0.914	12

*SD* Standard deviation, *min* Minimum, *max* Maximum, *FDG* ^18^F-fluordeoxyglucose, *AD-ROIs* Alzheimer’s disease signature regions of interest, *BL* At baseline, *PiB* ^11^C-Pittsburgh compound B, *global* Region of interest equals entire grey matter, *Δ* Changes between baseline and follow up adjusted to a 24 months follow up period, negative values indicate decrease, positive values indicate increase compared to BL

## Discussion

Post hoc power calculation revealed large effect sizes for PiB-PET and cortical thickness, and a smaller effect size for FDG-PET with regards to sensitivity to pick up AD progression over a pre-defined follow-up interval of 24 months in an early AD sample. Although true effect sizes tend to be overestimated in smaller samples and therefore minimum sample sizes would probably need to be larger than estimated based on our results, amyloid PET and cortical thickness require markedly smaller sample sizes compared to FDG-PET to monitor AD disease progression with surrogate biomarkers.

The strengths of our study include long-term follow-up data of three imaging biomarkers measured in parallel within a monocentric sample. Follow-up occurred after a predefined interval of 24 months. We specifically defined inclusion and exclusion criteria, biomarkers, and the interval between baseline and follow-up to match trials investigating disease modifying drugs in early AD. Hence, our results might contribute to selecting biomarkers and estimate sample sizes for tracking pathognomonic AD changes by surrogate biomarkers in future AD trials.

The strength of a monocentric sample might also be conceived as a limitation as clinical trials are multicentric. The biggest limitation, however, is the sample size. While the number of participants is sufficient for the applied statistics, results would be more robust using larger sample sizes from multiple centres. Although we aimed for the follow-up examination to take place after 24 months in some cases this was not obtainable. We adjusted for time deviations assuming a linear change which could be debated [42]. According to the model of Jack et al. biomarkers in AD change in a specific order over the course of the disease [42]. In this study, different measures typically showing more or less dynamic changes during the stage of early AD were assessed. At this point, it is important to keep in mind that we did not set out to compare how well suited the individual biomarkers are to support the diagnosis of AD pathology rather than to investigate how well they are suited to show progression in an early AD cohort such as it would be recruited for a clinical trial. In addition, one could argue that there might be better ways to analyse the imaging measures. Chen et al. and Su et al. both saw significantly greater power to detect Aβ increase on amyloid PET after 24 months when using cerebral-to white matter SUVR changes instead of using the cerebellum as reference region [18, 43]. Also other factors influencing the rate of amyloid accumulation such as white matter hyperintensities [44], baseline SUVR [45], and the status of markers for amyloid and neuronal injury at baseline [21] would have to be taken into account when planning biomarker assessments for a clinical trial. Chen et al. calculated minimal sample sizes for an assumed 12-month clinical trial. Comparable to the results of our study samples of *n* = 8 for Aβ positive subjects with probable Alzheimer’s dementia and *n* = 13 for Aβ positive subjects with MCI would be necessary to detect a 25% decrease of Aβ SUVR [18]. Considering the high sensitivity and specificity of FDG-PET to detect AD [46] we were surprised by its comparably small effect size regarding monitoring of disease progression. A possible explanation could be the method of evaluating FDG uptake. In contrast to amyloid PET where there is no or minimal specific tracer uptake in the reference region SUVRs for FDG-PET are obtained using a reference region with specific tracer uptake. Although reference regions chosen for FDG-PET are thought to be less affected by Alzheimer’s disease, in a complex network system like the brain, neuronal activity in one region might affect neuronal activity in other regions and thus could possibly affect uptake in the reference region and consequently SUVRs between baseline and follow-up. While results from dynamic tracer imaging might be more robust acquiring this data is more complex and necessitates invasive procedures such as arterial blood lines. Under the consideration of patients’ safety and feasibility it might not be a suitable approach during a clinical trial.

Lastly, the selection of and the confinement to imaging markers could be debated. Despite already comparing three different biomarkers in the same highly characterised cohort, other biomarkers such as tau-PET, CSF or blood based biomarkers might be even more powerful to monitor disease progression. Although we did not investigate any of the ^18^F-labeled tracers for Aβ it can be reasoned that results would be similar to the results we described for ^11^C labelled PiB-PET. However, this would have to be further investigated.

## Conclusion

When considering study designs amyloid imaging with PET and measuring cortical thickness with MRI are powerful biomarkers requiring relatively small sample sizes to monitor disease progression in early AD.

## Data Availability

The datasets generated during and/or analyzed during the current study are available from the corresponding author on reasonable request. However, due to the nature of pseudonymized patient data, a material transfer agreement is required to meet ethical standards and data privacy laws of Germany.

## Abbreviations

AD: Alzheimer’s disease
ADAS-cog: Alzheimer’s disease assessment scale – cognitive subscale
Aβ: Amyloid beta
C: Cerebrum
CDR-SOB: Clinical dementia rating scale sum of boxes
CSF: Cerebro spinal fluid
cv: Cerebellar vermis
FDG: ^18^F-fluordeoxyglucose
MCI: Mild cognitive impairment
MRI: Magnetic resonance imaging
PET: Positron emission tomography
PiB: ^11^C-Pittsburgh compound B
ROI: Region of interest
SUVR: Standardized uptake value ratio
