# Correction to: Amyloid PET, FDG-PET or MRI? - the power of different imaging biomarkers to detect progression of early Alzheimer’s disease

**DOI:** 10.1186/s12883-020-01649-9

**Published:** 2020-03-05

**Authors:** Marion Ortner, René Drost, Dennis Hedderich, Oliver Goldhardt, Felix Müller-Sarnowski, Janine Diehl-Schmid, Hans Förstl, Igor Yakushev, Timo Grimmer

**Affiliations:** 1 Department of Psychiatry and Psychotherapy, School of Medicine, Klinikum rechts der Isar, Technical University of Munich, Ismaninger Str. 22, 81675 Munich, Germany; 2 Department of Diagnostic and Interventional Neuroradiology, School of Medicine, Klinikum rechts der Isar, Technical University of Munich, Ismaninger Str. 22, 81675 Munich, Germany; 3 Department of Nuclear Medicine, School of Medicine, Klinikum rechts der Isar, Technical University of Munich, Ismaninger Str. 22, 81675 Munich, Germany

**Correction to: BMC Neurol**

**https://doi.org/10.1186/s12883-019-1498-9**

Following publication of the original article [1], the authors ask to correct the surname of co-author Dennis Hedderich from from He**ddd**erich to He**dd**erich.
